# Chronic myeloid leukemia presenting as an atraumatic splenic rupture in the setting of coronavirus disease 2019 infection

**DOI:** 10.1002/jha2.700

**Published:** 2023-04-28

**Authors:** Hanan Alharthy, Jaclyn Clark, Rima Koka, Ali Aldahmashi, Maria R. Baer

**Affiliations:** ^1^ Department of Medicine University of Maryland School of Medicine Baltimore Maryland USA; ^2^ University of Maryland Greenebaum Comprehensive Cancer Center Baltimore Maryland USA; ^3^ Department of Surgery University of Maryland School of Medicine Baltimore Maryland USA; ^4^ R Adams Cowley Shock Trauma Center the University of Maryland School of Medicine Baltimore Maryland USA; ^5^ Department of Pathology University of Maryland School of Medicine Baltimore Maryland USA; ^6^ Department of Diagnostic Radiology and Nuclear Medicine University of Maryland School of Medicine Baltimore Maryland USA

**Keywords:** chronic myeloid leukemia, COVID‐19, hyperleukocytosis, splenic rupture

1

A 68‐year‐old woman developed pharyngitis and had a positive coronavirus disease 2019 (COVID‐19) home antigen test. Four days later, she presented to the emergency department with sudden severe diffuse abdominal pain radiating to her left shoulder and back. She denied fall or trauma. Her abdomen was diffusely tender. Hemoglobin was 9.6 g/dl, white blood cell (WBC) count 132 × 10^9^/L with 48% segmented neutrophils, 19% bands, 3% lymphocytes, 3% monocytes, 7% basophils, 5% metamyelocytes, 14% myelocytes, and 1% blasts, and platelet count 515 × 10^9^/L. Computed tomography angiography showed a 17.9 cm spleen with evidence of laceration and intraparenchymal active bleeding, associated with a moderate volume hemoperitoneum with high‐intensity blood products adjacent to the spleen and layering in the pelvis (Figure 1). She developed hemorrhagic shock and worsening respiratory status requiring intubation. Emergent exploratory laparotomy and splenectomy were performed. One liter of blood was evacuated from her abdomen, and a 950‐gram spleen (normal weight, 150 g) was removed. Microscopy showed areas of capsular disruption with underlying hemorrhage and necrosis, with involvement by chronic myeloid leukemia (CML) without increased blasts (Figure 2). Bone marrow biopsy was more than 90% cellular with myeloid predominance, also without increased blasts (Figure 3). Karyotype was 46,XX,t(9;22)(q34;q11.2) in 20 metaphases, and BCR::ABL1 fusion transcripts [e13a2 (b2a2)] were detected by reverse transcription‐polymerase chain reaction. BCR::ABL1/ABL1% (IS) was 71.5505%. CML in the chronic phase was diagnosed. She had a EUTOS long‐term survival score of 1.6475 (estimating spleen size below costal margin), placing her in the intermediate‐risk group [1]. Treatment with the BCR::ABL inhibitor dasatinib was initiated at a dose of 100 mg daily on postoperative day 6. Platelet count increased after splenectomy, reaching a maximum of 1351 × 10^9^/L on postoperative day 16. WBC and platelet counts decreased to normal values on day 24 of dasatinib therapy. She continues treatment with dasatinib and is tolerating it well. BCR::ABL1/ABL1% 6 months after treatment initiation was 0.056%. Blood counts remain normal at nine months.

**FIGURE 1:**
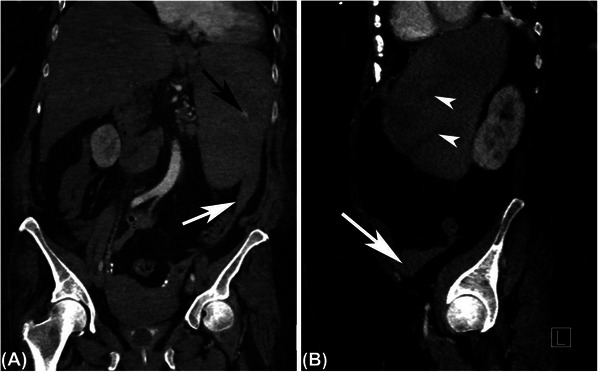
Contrast‐enhanced computed tomography. Coronal (A) and Sagittal (B) views of the abdomen and pelvis demonstrate a small area of contrast extravasation (black arrow) in an enlarged, heterogeneous spleen with multiple hypodense lines suggestive of lacerations (white arrowheads). Hemoperitoneum was also evident (white arrows).

**FIGURE 2:**
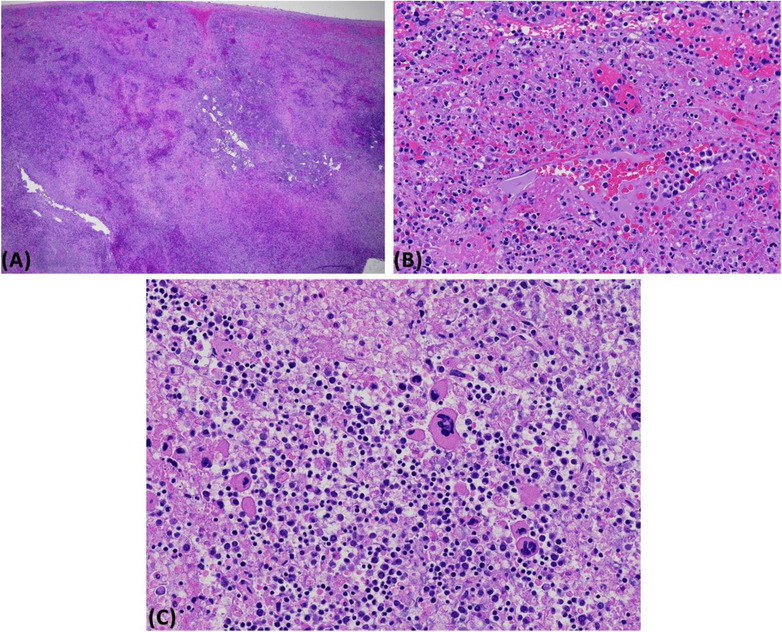
Hematoxylin and eosin‐stained sections of the 950‐g spleen, (A) x20 magnification, (B) x200 magnification, show areas of capsular disruption with underlying hemorrhage and necrosis. The viable areas, (C) x200 magnification, showed myeloid cells without increased blasts and with atypical hypolobated megakaryocytes, compatible with involvement by chronic myeloid leukemia in the chronic phase.

**FIGURE 3:**
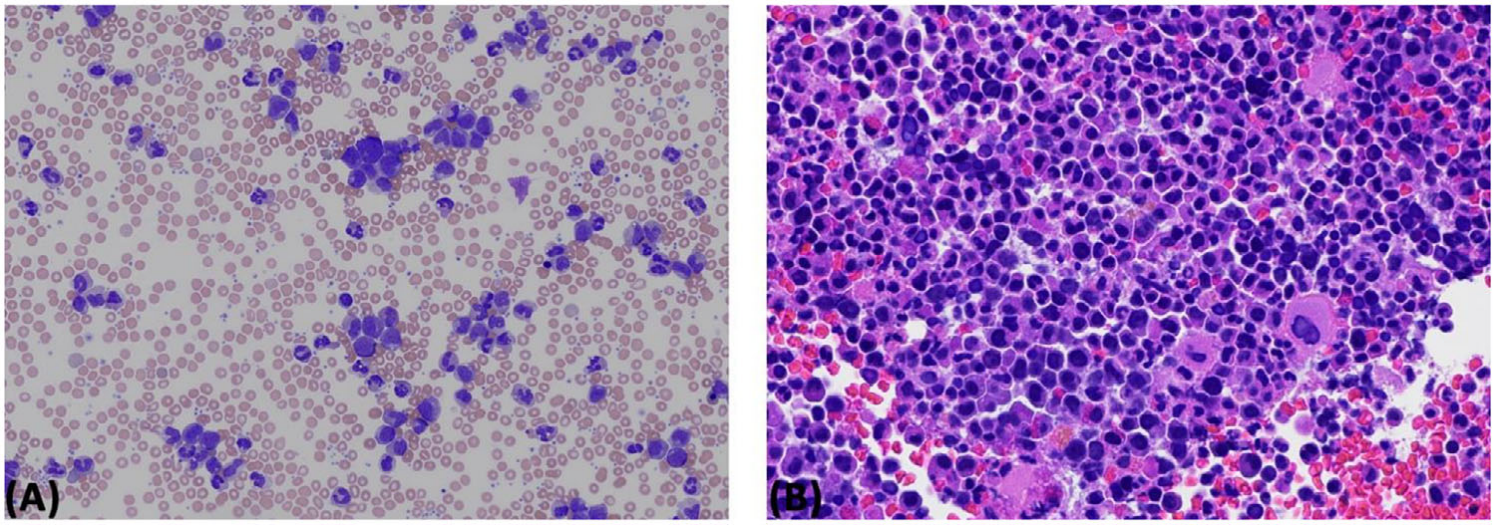
Hematoxylin and eosin‐stained peripheral blood smear, (A) x100 magnification, shows prominent leukocytosis with left‐shifted neutrophils, and bone marrow core biopsy, (B) x400 magnification, shows a markedly hypercellular marrow composed of maturing neutrophil precursors and hypolobated megakaryocytes.

Atraumatic splenic rupture is a rare and frequently fatal event that occurs mostly in the setting of underlying pathological conditions, including infections and malignancies, both hematologic and non‐hematologic. Non‐Hodgkin lymphoma accounts for most neoplastic causes, followed by acute leukemias and, less commonly, CML and myelofibrosis [2, 3, 4]. Of all cases with atraumatic splenic rupture secondary to hematologic malignancies, CML accounts for up to 18% [3]. Proposed mechanisms for splenic rupture in hematologic malignancies include direct infiltration of the spleen by malignant cells leading to an increase in volume and stretching of the relatively non‐expandable capsule, ultimately resulting in capsular rupture [4], as well as infarction and coagulation defects leading to spontaneous intraparenchymal splenic bleeding and subsequent capsule rupture [4].

Spontaneous splenic rupture without underlying spleen pathology has been reported in the setting of COVID‐19 infection [5]. The severe acute respiratory syndrome coronavirus 2 virus elicits intense inflammation that affects many organ systems. It enters respiratory endothelial cells via the angiotensin‐converting enzyme 2 receptors, leading to endothelial activation and increased angiotensin II levels [6, 7]. Endothelial activation is an essential step that triggers a cascade of events including: (1) increased von Willebrand factor, causing platelet adhesion and aggregation, (2) secretion of plasminogen activator inhibitor‐1, leading to fibrinolysis, (3) expression of tissue factor, causing activation of the coagulation cascade, and (4) secretion of the pro‐inflammatory interleukins (ILs) IL‐1 and IL‐6. Additionally, increased angiotensin II levels further activate the coagulation cascade [7]. These changes lead to an inflammatory and prothrombotic state which can cause microthrombi in diverse organs, including the spleen, predisposing patients to splenic rupture [5, 8]. Patients with COVID‐19 infections have also been found to have splenic white pulp atrophy due to lymphocyte depletion, in addition to areas of hemorrhage and congestion [9].

To our knowledge, this is the first reported case of atraumatic splenic rupture in the setting of COVID‐19 infection in a patient with undiagnosed underlying CML. It is likely that our patient had splenomegaly due to CML, which predisposed to spontaneous splenic rupture in the setting of her COVID‐19 infection, due to further stretching of the splenic capsule caused by endothelial dysregulation, cytokine release, increased vascular permeability, and congestion, causing a spontaneous subcapsular hematoma that led to capsule disruption and rupture.

Patients with hematologic malignancies are at increased risk for complications of COVID‐19 infection. Although extremely rare, spontaneous splenic rupture in these patients can lead to a fatal outcome. Prompt identification and management of this serious complication prevent mortality.

## AUTHOR CONTRIBUTIONS

Hanan Alharthy, Jaclyn Clark, Rima Koka, Ali Aldahmashi, and Maria R. Baer performed the research.

Hanan Alharthy and Maria R. Baer wrote the paper.

## FUNDING INFORMATION

Supported by National Cancer Institute Cancer Center Support Grant (CCSG) P30CA134274.

## CONFLICT OF INTEREST STATEMENT

The authors have no actual or potential conflicts of interest relevant to this manuscript.

## ETHICS STATEMENT

The University of Maryland School of Medicine does not require Institutional Review Board review and approval of a deidentified case report of a single patient.

## PATIENT CONSENT STATEMENT

N/A

## CLINICAL TRIAL REGISTRATION

N/A

## Data Availability

Data sharing is not applicable to this article as no datasets were generated during the current study.
